# Combination of transcatheter arterial chemoembolization and interrupted dosing sorafenib improves patient survival in early–intermediate stage hepatocellular carcinoma: A post hoc analysis of the START trial

**DOI:** 10.1097/MD.0000000000007655

**Published:** 2017-09-15

**Authors:** Teng-Yu Lee, Chen-Chun Lin, Chiung-Yu Chen, Tsang-En Wang, Gin-Ho Lo, Chi-Sen Chang, Yee Chao

**Affiliations:** a Division of Gastroenterology and Hepatology, Department of Internal Medicine, Taichung Veterans General Hospital; b Department of Medicine, Chung Shan Medical University, Taichung; c Division of Hepatology, Liver Research Unit, Department of Gastroenterology and Hepatology, Linkou Chang Gung Memorial Hospital–Chang Gung University, Linkou; d National Cheng Kung University Hospital, College of Medicine, National Cheng Kung University, Tainan; e Mackay Memorial Hospital, Taipei; f Department of Medical Research, E-Da Hospital, School of Medicine for International Students, I-Shou University, Kaohsiung; g Department of Oncology, Taipei Veterans General Hospital, National Yang-Ming University, Taipei, Taiwan.

**Keywords:** hepatoma, molecular targeted therapy, patient outcome, therapeutic embolization

## Abstract

**Background/Objective:**

The survival benefit of treatment for unresectable hepatocellular carcinoma (HCC) with transcatheter arterial chemoembolization (TACE) combined with sorafenib remains uncertain. We compared the survival of patients treated with TACE and sorafenib with that of patients treated with TACE alone.

**Methods:**

This was a post hoc analysis of the Study in Asia of the Combination of TACE with Sorafenib in Patients with HCC (START) trial. All patients who received TACE and interrupted dosing of sorafenib for early or intermediate-stage HCC in Taiwan from 2009 to 2010 were recruited into the TACE and sorafenib group. They were randomly matched 1:1 by age, sex, Child–Pugh score, tumor size, tumor number, and tumor stage with patients from Taichung Veterans General Hospital in Taiwan who received TACE alone and who fulfilled the selection criteria of the START trial during the same time period (control group). Patient survival [cumulative incidence and hazard ratio (HR)] of the 2 groups were analyzed and compared.

**Results:**

The baseline characteristics of the 36 patients in each group were similar. Tumor response rates were significantly better in the TACE and sorafenib group (*P* < .04). Overall survival of the TACE and sorafenib group was also significantly better than that of the control (TACE alone) group over the 2 years [78%, 95% confidence interval (95% CI) 64–91 vs 49, 95% CI 32–66; *P* = .012]. In the multivariate regression analysis, TACE and sorafenib was found to be independently associated with a decreased risk of mortality (HR 0.33, 95% CI 0.12–0.89; *P* = .015). Multivariate stratified analyses verified this association in each patient subgroup (all HR < 1.0).

**Conclusion:**

With a high patient tolerance to an interrupted sorafenib dosing schedule, the combination of TACE with sorafenib was associated with improved overall survival in early–intermediate stage HCC when compared with treatment with TACE alone.

## Abstract 2

Supplemental Digital Content is available in the text

## 1 Introduction

Hepatocellular carcinoma (HCC) is a highly fatal malignancy and remains one of the leading causes of cancer death worldwide.^[1]^ Even with progress in HCC surveillance, only a small proportion of new cases can receive curative treatment; potentially palliative treatments, such as transcatheter arterial chemoembolization (TACE), are frequently used for unresectable HCC.^[2,3]^ Under current practice guidelines, TACE is the recommended standard of care for intermediate-stage HCC.^[4–6]^ Moreover, TACE is used to treat early-stage HCC patients in whom curative treatment has failed or is infeasible.^[7]^ However, the clinical outcomes of patients who receive TACE remain unsatisfactory. In previous randomized controlled trials (RCTs) wherein patients with good liver condition were strictly selected, the 3-year survival rates of the TACE groups were only around 30%.^[8,9]^ There is a great need to improve the survival rates of patients who receive TACE.

Sorafenib has been shown to be successful in improving patient survival in advanced-stage HCC,^[10,11]^ so some experts have reasonably hypothesized that the combination of sorafenib with TACE for the treatment of locoregional HCCs would improve survival.^[12]^ However, the survival benefit of this combination strategy has not been demonstrated in RCTs.^[13]^ With the discrepancy between the hypothesis and previous trial results on the TACE and sorafenib combination, concerns related to study heterogeneity in previous clinical trials have been raised, and a positive result demonstrating clear survival benefit from a clinical trial is still pending.^[14]^

In our recently published Phase II clinical trial [Study in Asia of the Combination of TACE with Sorafenib in Patients with HCC (START)], we showed that the combination of TACE and sorafenib is not only well tolerated but also efficacious.^[15]^ An interrupted sorafenib dosing schedule helped to reduce the number of adverse events caused by the combination treatment, and even improved patient compliance and clinical outcomes. However, a comparator arm in which patients received TACE alone was lacking in the START trial. We therefore conducted a post hoc analysis to compare patient survival in the TACE and sorafenib group and a matched control TACE alone group.

## 2 Methods

### 2.1 Study design and participants

We conducted a post hoc analysis of our recently published clinical trial (START; ClinicalTrials.gov registration #NCT00990860).^[15]^ START was a Phase II, investigator-initiated, prospective, single-arm multinational study that evaluated sorafenib in combination with TACE in patients with locoregional HCC. Patients were enrolled from 2009 to 2010. A detailed description of the study methods, and inclusion and exclusion criteria, are available in the previously published report by Chung et al.^[16]^

In this post hoc analysis, all Taiwanese patients with early or intermediate-stage HCC in the START trial were recruited into a TACE and sorafenib group. They were then randomly matched 1:1 by age, sex, Child–Pugh score, tumor size, tumor number, and tumor stage with patients from Taichung Veterans General Hospital in Taiwan who received TACE alone and who fulfilled the patient selection criteria of the START trial during the same time period (control group). Patients with advanced HCC were excluded from this present study. The clinical outcomes of the TACE and sorafenib group and the control group were compared. This study was conducted in accordance with the principles in the Declaration of Helsinki on human research. Approval from the institutional review board of Taichung Veterans General Hospital (No. CE14263A) was also obtained for this study.

### 2.2 TACE procedure

Conventional TACE was performed by experienced radiologists. The feeding vessels of tumors were selectively catheterized to preserve as much of the liver parenchyma as possible. Transarterial chemotherapy was performed using a mixture of lipiodol and a chemotherapeutic agent. Afterwards, embolization with absorbable particles (Gelfoam; Pfizer Inc., New York, NY) was done until complete flow stagnation was achieved. The detailed procedures which we followed for TACE were as previously described.^[16]^ Dynamic computed tomography (or, alternatively, magnetic resonance imaging) of the abdomen with contrast agent administration was used to evaluate the need for subsequent TACE 4 to 8 weeks after the index TACE. TACE was repeated on demand according to clinician judgment. If no viable tumor was found on dynamic imaging study, follow-up computed tomography (or magnetic resonance imaging) was arranged at 3-month intervals. If dynamic imaging study revealed new lesions, the patient was evaluated to determine whether new TACE treatment would be feasible.

### 2.3 The interrupted schedule of sorafenib therapy

In the START trial, patients were initially started on sorafenib therapy (400 mg BID) on day 4 (to day 7) after the index TACE (day 1). If a follow-up imaging study showed viable HCC, another course of TACE would be scheduled. Sorafenib therapy would be interrupted after the evening dose on day 4 before each subsequent TACE, and sorafenib therapy would be restarted on day 4 (to day 7) after each subsequent TACE. If patients did not receive further TACE, then they would receive continuous sorafenib therapy. The detailed schedules of sorafenib therapy were as previously described.^[16]^ The maximum duration of sorafenib treatment was 2 years after the index TACE. Sorafenib was used until stage migration during the study period. In contrast, none of the patients in the control (TACE alone) group received sorafenib therapy during the study period.

### 2.4 Outcome measures

We used modified RECIST (Response Evaluation Criteria In Solid Tumors) criteria to measure tumor size and tumor response.^[17]^ Apart from patients with extrahepatic metastasis, a maximum of 2 lesions in the liver were designated as target lesions by dynamic imaging study at the time of assessment. Tumor response was evaluated after comprehensively examining target lesions and nontarget lesions within or outside the liver. Tumor responses were classified as complete response (CR), partial response (PR), stable disease (SD), or progressive disease (PD) according to the modified RECIST criteria. We also evaluated time to progression (TTP) and overall survival (OS).

### 2.5 Statistical analysis

Using a logistic model with age, sex, Child–Pugh score, tumor size, tumor number, and tumor stage, propensity scores were measured and patients in the 2 groups were matched. Data of continuous variables are presented as mean ± standard deviation and median (interquartile range). Data of discrete variables are presented as number (%). Continuous variables were compared using the Mann–Whitney *U* test, and discrete variables were compared using Fisher exact test. Cumulative incidences for time-to-event (tumor progression or patient mortality) were calculated, and death before tumor progression was considered a competing risk event.^[18]^ Cumulative incidences of the TACE and sorafenib group and the control (TACE alone) group were compared using a modified Kaplan–Meier method, and differences in the full time-to-event distributions were compared using a modified log-rank test. After adjusting for age, sex, serum bilirubin level, serum albumin level, Child–Pugh score, tumor size, tumor number, Barcelona Clinic Liver Cancer (BCLC) tumor stage, and serum alpha-fetoprotein (AFP) level, multivariate regression analyses were conducted to determine the independent prognostic factors for OS. Hazard ratios (HRs) were also determined by means of a modified Cox proportional hazard model in the presence of competing risk events. A *P* value of less than .05 was considered to be statistically significant. Data analyses were carried out using IBM SPSS Statistics for Windows, Version 20.0 (IBM Corp., Armonk, NY).

## 3 Results

### 3.1 Baseline demographic characteristics

There were 36 patients with early or intermediate-stage HCC in the TACE and sorafenib group, and 36 patients with early or intermediate-stage HCC in the TACE-alone control group. As summarized in Table 1, the baseline demographic data of patients in both groups were similar. The majority of patients in both groups were male (> 80%) and in late middle age. Liver function was generally well compensated, and the mean and median values of serum bilirubin and albumin were within normal limits. The median Child–Pugh score of each group was 5.0. Tumor burden, including maximum tumor size and tumor number, was also similar in both groups. Although the proportion of patients in BCLC stage A was slightly lower in the TACE and sorafenib group (25.0%) than in the control group (41.7%), the difference was not statistically significant (*P* = .21). The mean and median values of serum AFP were also similar in both groups. In the TACE and sorafenib group, the mean daily dose of sorafenib was 660.6 ± 152.0 mg (median, 752.8 mg), and the mean duration of sorafenib treatment was 378.2 ± 223.7 days (median, 394.0 days).

**Table 1:**
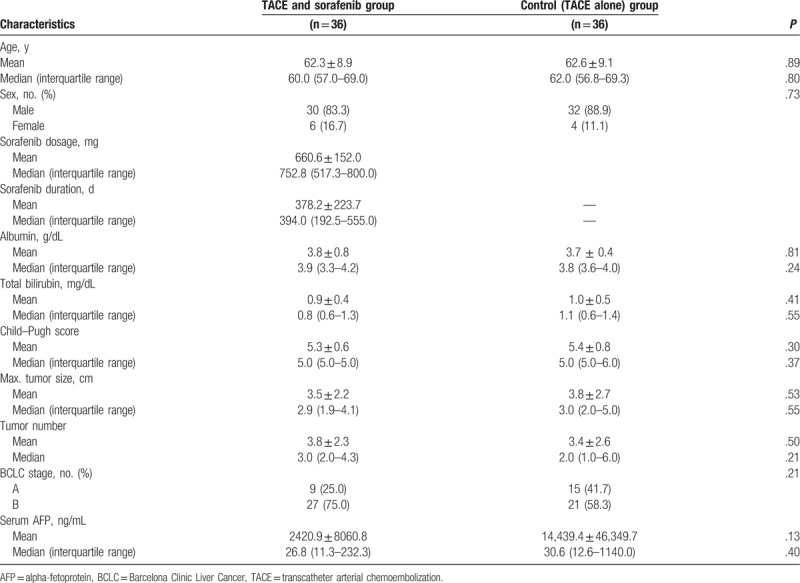
Baseline characteristics of study participants.

### 3.2 Tumor responses and time to progression

Radiological responses were evaluated by modified RECIST criteria. The tumor responses observed in the TACE and sorafenib group were significantly better than those in the control group (CR, 55.6% vs 33.3%; PR, 33.3% vs 38.9%; SD, 11.1% vs 11.1%; PD: 0% vs 16.7%; *P* = .04). The disease control rate (CR + PR + SD) of the TACE and sorafenib group was significantly higher than that of the control group (100.0% vs 83.3%, *P* = .03). However, although median TTP in the TACE and sorafenib group was longer than that in the control group, the difference was not statistically significant [0.65 (0.44–1.08) vs 0.38 (0.21–0.95) years; *P* = .14].

### 3.3 Overall survival

**Figure 1:**
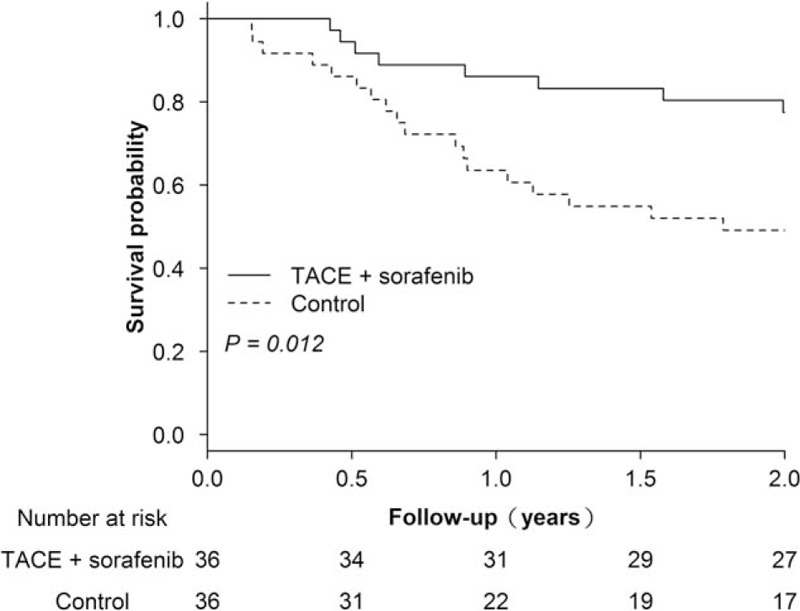
Overall survival of patients with early–intermediate stage hepatocellular carcinoma in the TACE and sorafenib group and the control (TACE alone) group over the 2-year study period. TACE = transcatheter arterial chemoembolization.

As shown in Fig. 1, the 2-year OS in the TACE and sorafenib group was significantly higher than that of the control group [1-year survival probabilities: 0.86 (95% confidence interval, 95% CI, 0.75–0.98) vs 0.64 (95% CI, 0.47–0.80); 2-year survival probabilities: 0.78 (95% CI, 0.64–0.91) vs 0.49 (95% CI, 0.32–0.66); *P* = .012]. Furthermore, TACE and sorafenib combination (HR, 0.35; 95% CI, 0.16–0.81), Child–Pugh score (HR, 2.21; 95% CI, 1.39–3.51), maximum tumor size (HR, 1.34; 95% CI, 1.18–1.52), BCLC stage A (HR, 0.31; 95% CI, 0.11–0.91), serum AFP > 200 ng/mL (HR, 2.77; 95% CI, 1.29–5.95), and tumor responder (CR + PR) (HR, 0.43; 95% CI, 0.19–0.97) were associated with improved patient survival in the univariate regression analysis (Table 2). However, in the multivariate regression analysis, only TACE and sorafenib combination (HR, 0.35; 95% CI, 0.15–0.81), Child–Pugh score (HR, 1.84; 95% CI, 1.09–3.09), maximum tumor size (HR, 1.29; 95% CI, 1.10–1.52), and serum AFP >200 ng/mL (HR, 2.55; 95% CI, 1.08–6.00) were independent prognostic factors for patient survival.

**Table 2:**
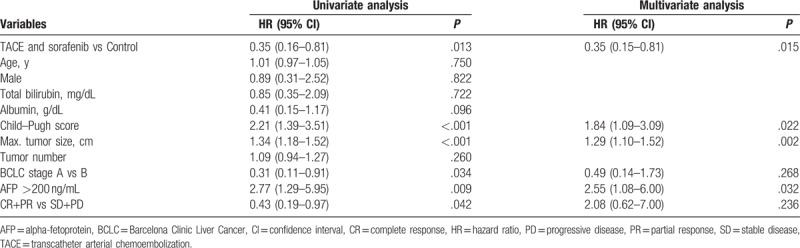
Cox proportional hazard model analysis for overall survival.

**Figure 2:**
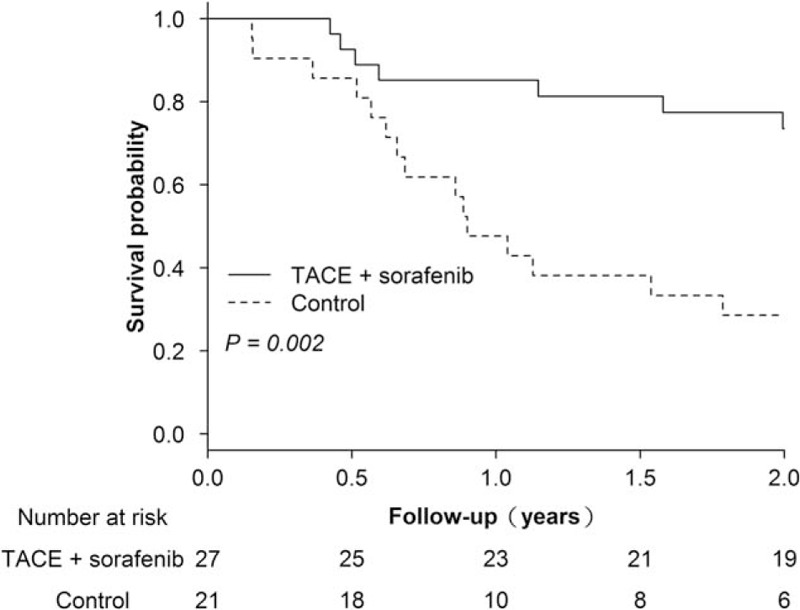
Overall survival of patients with intermediate stage hepatocellular carcinoma in the TACE and sorafenib group and the control (TACE alone) group over the 2-year study period. TACE = transcatheter arterial chemoembolization.

We also analyzed OS among patients with intermediate-stage HCC. As shown in Fig. 2, the 2-year OS was significantly higher in the TACE and sorafenib group than in the control group [1-year survival probabilities: 0.85 (95% CI, 0.72–0.99) vs 0.48 (95% CI, 0.25–0.70); 2-year survival probabilities: 0.74 (95% CI, 0.56–0.91) vs 0.29 [95% CI, 0.08–0.49); *P* = .002].

Interestingly, after the end of the 2-year sorafenib treatment in the START trial (and patients were no longer given sorafenib), OS in the TACE and sorafenib group at the 3-year mark was not significantly different from that of the control group [3-year survival probabilities: 0.42 (95% CI, 0.25–0.59) vs 0.38 (95% CI, 0.21–0.54); *P* = .245; see Figure, Supplemental Content].

### 3.4 Multivariate stratified analyses in subgroups of patients

**Figure 3:**
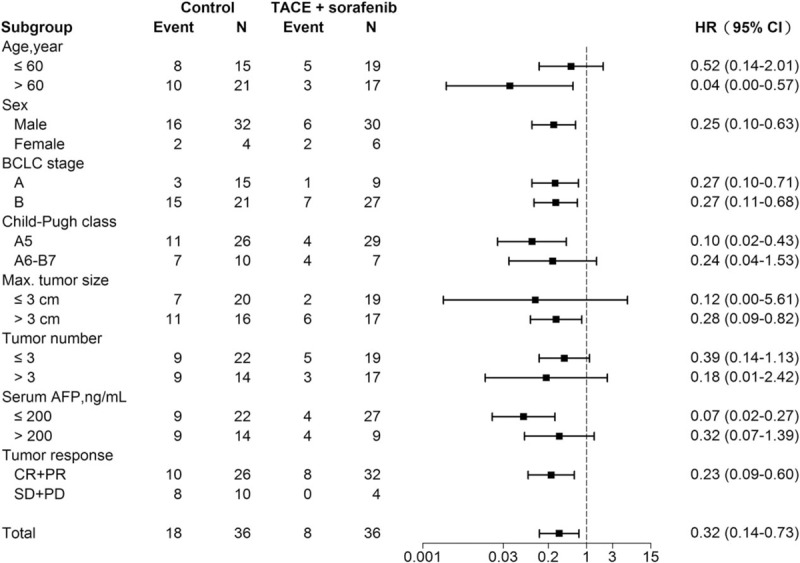
Multivariate stratified analysis of the association between TACE and sorafenib combination treatment and mortality risk. AFP = alpha-fetoprotein, BCLC = Barcelona Clinic Liver Cancer, CI = confidence interval, CR = complete response, HR = hazard ratio, PD = progressive disease, PR = partial response, SD = stable disease.

Multivariate stratified analyses for survival benefit of TACE and sorafenib combination therapy was performed in subgroups of patients (subgroups shown in Fig. 3). TACE and sorafenib combination therapy was associated with decreased overall mortality in each patient subgroup (all HR <1.0). Furthermore, regarding the association between TACE and sorafenib combination and reduced overall mortality risk, statistical significance was reached in some subgroups (age >60 years, male sex, BCLC stage A or B, Child–Pugh class A5, tumor size >3 cm, AFP ≤200 ng/mL, and tumor response CR and PR).

### 3.5 Discussion

Although some Phase II clinical trials have shown encouraging results that TACE combined with sorafenib could be efficacious in the management of locoregional HCC,^[15,19,20]^ RCTs have failed to demonstrate any survival benefit from combined therapy.^[13,21]^ However, variations in treatment regimens, such as the timing of sorafenib discontinuation or repeating TACE, have been observed in different regions.^[21,22]^ In 1 study, Asian patients appeared to have a greater survival benefit from TACE and sorafenib than non-Asian patients.^[21]^ It is also possible that TACE operators in different regions may contribute to differences in outcome.^[23]^ In this study, because patients were recruited from the same region, heterogeneity in the treatment process may be reduced. We demonstrated that patient survival was improved by TACE and sorafenib compared with TACE alone. Our findings provide a basis for conducting well-designed RCTs in the future.

On the basis of previous RCTs of TACE and sorafenib for locoregional HCC, sustaining sorafenib therapy for a sufficient period of time has been considered an important prognostic factor.^[14]^ For example, compared with non-Asian patients in the SPACE trial, longer duration of sorafenib treatment was associated with longer OS in Asian patients (median duration 30 vs 17 weeks). Similarly, compared with Japanese patients in the Japan–Korea trial, longer duration of sorafenib treatment was associated with longer TTP in Korean patients (median duration 31 vs 16 weeks). In this study, the median duration of sorafenib therapy was 394 days (56 weeks); the good survival benefit we observed may have been related to the lengthy duration of sorafenib treatment. According to the START findings, an interrupted sorafenib dosing schedule (in which sorafenib administration was stopped for 4 days before and after TACE) contributed to considerably fewer adverse events than observed in other trials of combination therapy; this dosing strategy may help patients tolerate a longer duration of sorafenib therapy.

Tumor response was significantly better among patients who received TACE and sorafenib than among those who received TACE alone in this study. Improved tumor response rates in the TACE and sorafenib group may also have contributed to better patient survival. Due to the hypoxic effect in tumor cells induced by arterial embolization, TACE can result in a rapid release of tumor neovascularization mediators such as vascular endothelial growth factor (VEGF).^[24,25]^ VEGF level has been found to be an independent prognostic factor in patients with unresectable HCC.^[25,26]^ Sorafenib not only inhibits tumor proliferation but also prevents tumor neoangiogenesis by blocking VEGF receptors.^[27,28]^ In our previous investigation of HCC patients who received TACE, serum VEGF level progressively increased, peaking on day 14 after TACE.^[29]^ There are good reasons to support the use of potent multikinase inhibitors (such as sorafenib) after TACE to improve clinical outcomes.

We found that the survival benefit of TACE and sorafenib combination therapy seemed to diminish after the discontinuation of sorafenib. This phenomenon may be seen as inverse evidence supporting the use of sorafenib. The optimal timing of sorafenib discontinuation remains under debate.^[30–32]^In a mouse model evaluating the impact of sorafenib discontinuation,^[33]^ it was found that transient sorafenib interruption did not impede restoration of tumor response, but definitive sorafenib interruption tended to stimulate a rebound in angiogenesis to a higher level than if sorafenib treatment had never been given. In a retrospective cohort study of patients with advanced HCC,^[31]^ where even radiologic PD had been confirmed, continuing with sorafenib therapy improved patient survival compared with sorafenib discontinuation. Our findings suggest that sorafenib therapy should be continued for as long as possible, but a prospective study is needed for confirmation.

Several limitations should be acknowledged in this study. First, selection bias might potentially exist in this retrospective post hoc analysis, though we have tried to minimize this possibility by matching all possible confounders (such as age, sex, Child–Pugh score, tumor size, tumor number, tumor stage) between the TACE and sorafenib group and the control group. Second, the number of cases in this study was not large, so we used multivariate and subgroup analyses to examine our findings. Nevertheless, a large-scale study should be performed for confirmation. Last, although the difference was not statistically significant, the proportion of patients in BCLC stage A was slightly higher in the control group. However, the clinical outcome of HCC patients in the early stage is theoretically better than that of patients with intermediate-stage HCC, so the observation that TACE and sorafenib combination therapy could improve patient survival remains unchanged.

In conclusion, with high patient tolerance to an interrupted sorafenib dosing schedule, the combination of TACE and sorafenib was associated with improved OS in early–intermediate stage HCC compared with TACE alone.

## Supplementary Material

Supplemental Digital Content
