# Supplementary material for: Combination of transcatheter arterial chemoembolization and interrupted dosing sorafenib improves patient survival in early–intermediate stage hepatocellular carcinoma: A post hoc analysis of the START trial
Source: Medicine (Baltimore). 2017 Sep 15;96(37):e7655. doi: 10.1097/MD.0000000000007655 (PMC5604624; doi:10.1097/MD.0000000000007655)

**Supplemental Content**

**Figure.** Overall survival of patients with early–intermediate stage hepatocellular carcinoma in the TACE + sorafenib group and the control (TACE alone) group over 3 years. TACE = transcatheter arterial chemoembolization.


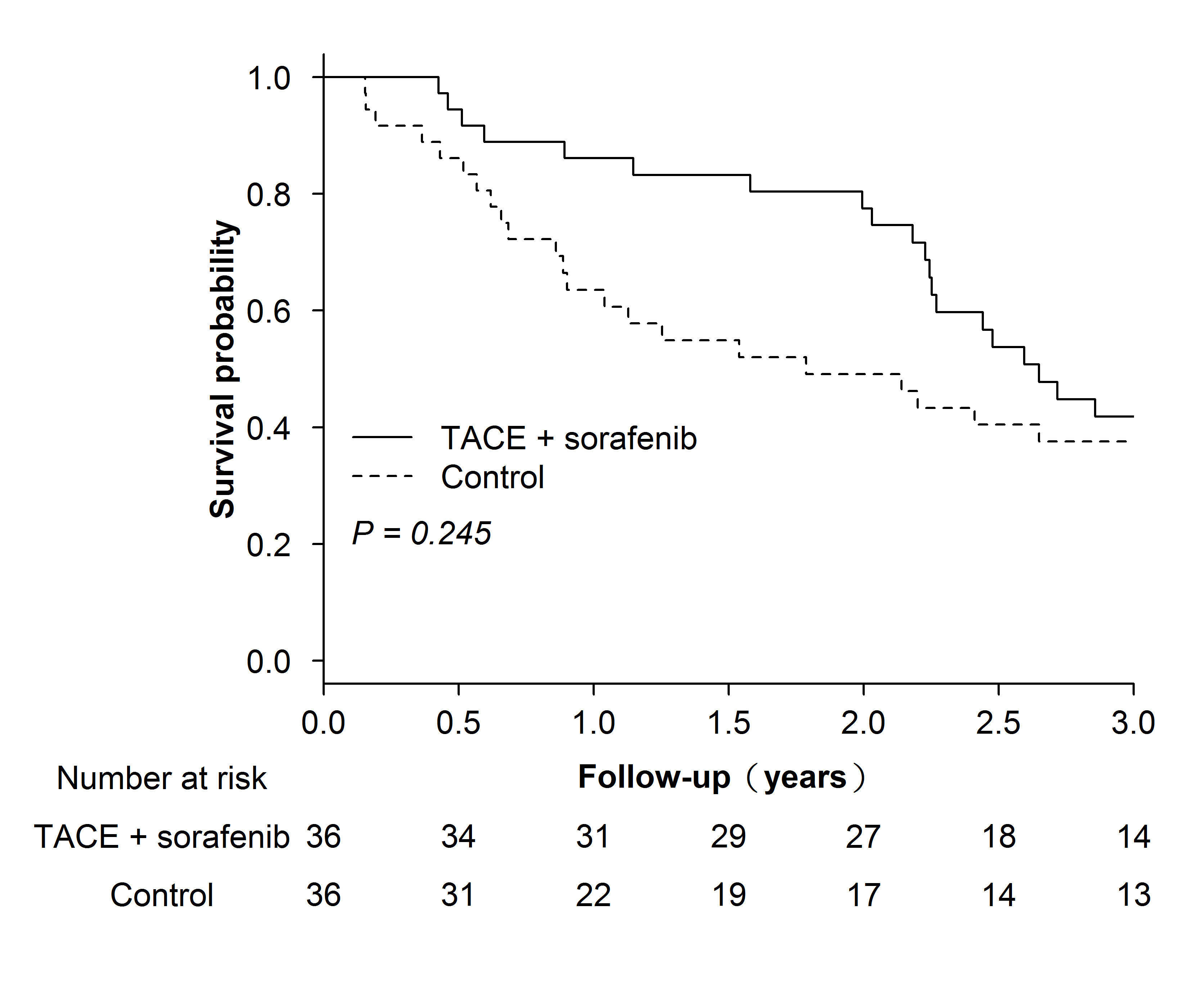

Supplement: Supplemental Digital Content [file medi-96-e7655-s001.doc]
